# Distinct Early Molecular Responses to Mutations Causing vLINCL and JNCL Presage ATP Synthase Subunit C Accumulation in Cerebellar Cells

**DOI:** 10.1371/journal.pone.0017118

**Published:** 2011-02-17

**Authors:** Yi Cao, John F. Staropoli, Sunita Biswas, Janice A. Espinola, Marcy E. MacDonald, Jong-Min Lee, Susan L. Cotman

**Affiliations:** Molecular Neurogenetics Unit, Center for Human Genetic Research, Massachusetts General Hospital, Boston, Massachusetts, United States of America; Tokyo Medical and Dental University, Japan

## Abstract

Variant late-infantile neuronal ceroid lipofuscinosis (vLINCL), caused by *CLN6* mutation, and juvenile neuronal ceroid lipofuscinosis (JNCL), caused by *CLN3* mutation, share clinical and pathological features, including lysosomal accumulation of mitochondrial ATP synthase subunit c, but the unrelated *CLN6* and *CLN3* genes may initiate disease via similar or distinct cellular processes. To gain insight into the NCL pathways, we established murine wild-type and Cb*Cln6* ^nclf/nclf^ cerebellar cells and compared them to wild-type and Cb*Cln3* ^Δex7/8/Δex7/8^ cerebellar cells. Cb*Cln6* ^nclf/nclf^ cells and Cb*Cln3* ^Δex7/8/Δex7/8^ cells both displayed abnormally elongated mitochondria and reduced cellular ATP levels and, as cells aged to confluence, exhibited accumulation of subunit c protein in Lamp 1-positive organelles. However, at sub-confluence, endoplasmic reticulum PDI immunostain was decreased only in Cb*Cln6* ^nclf/nclf^ cells, while fluid-phase endocytosis and LysoTracker® labeled vesicles were decreased in both Cb*Cln6* ^nclf/nclf^ and Cb*Cln3* ^Δex7/8/Δex7/8^ cells, though only the latter cells exhibited abnormal vesicle subcellular distribution. Furthermore, unbiased gene expression analyses revealed only partial overlap in the cerebellar cell genes and pathways that were altered by the *Cln3* ^Δex7/8^ and *Cln6* ^nclf^ mutations. Thus, these data support the hypothesis that *CLN6* and *CLN3* mutations trigger distinct processes that converge on a shared pathway, which is responsible for proper subunit c protein turnover and neuronal cell survival.

## Introduction

The neuronal ceroid lipofuscinoses (NCLs) collectively account for most cases of childhood-onset neurodegenerative disease worldwide, with clinical features of blindness, seizures, psychosis, motor and cognitive decline, and premature death (see recent reviews [1], [2]). A defining feature of NCL is lysosomal storage of autofluorescent ceroid lipofuscin, which contains proteolipid and dolichols, and in most forms of NCL, the mitochondrial ATP synthase, subunit c protein [2], [3], [4], [5].

Childhood-onset NCL is recessively inherited, and rare recessive and dominant adult-onset forms of NCL have also been described [2], [6], [7], [8]. To date, 10 genetic loci are linked to NCL, and it is likely more are yet to be discovered [2], [6]. The genes linked to NCL encode proteins primarily localized to either acidic organelles (late endosomes and lysosomes) or to the endoplasmic reticulum (ER). Several of the proteins are enzymes (PPT1, TPPI, cathepsin D), but the others are novel, mostly transmembrane proteins, with no known function (see recent reviews [2], [6], [9]).

Given the overlapping clinical symptoms and disease pathology in the different forms of NCL, it has been proposed that the NCL genes encode proteins that function together or at different points in a common pathway, which most likely involves lipid and protein trafficking pathways and/or ion homeostasis [2], [9]. Consistent with this hypothesis, protein-protein interaction between several NCL-linked proteins has been implicated by studies in overexpression or pull-down assay systems [10], [11], and cross-correction of growth defects in patient cells by other *CLN* genes has been described [12]. However, clinical and pathological differences in the different NCL sub-types are also recognized, including distinctive ultrastructure of the storage material and differences in the age at onset and order of symptom onset [1], [13], [14], [15].

The most common form of NCL, with juvenile onset (JNCL), is caused by *CLN3* mutation [16]. The *CLN3* gene encodes a novel multipass transmembrane protein (battenin or CLN3p) that primarily localizes to the late endosome and lysosome in most cell types. CLN3p is implicated in regulation of lysosomal pH [17], [18], endocytosis [19], [20], [21], autophagy [22], [23], cell growth and survival [24], [25], palmitoyl desaturase activity [26], and lysosome-targeted protein trafficking [20], [27]. However, the precise protein activity of CLN3p remains unknown.

*CLN6* mutation causes a non-classical, hence ‘variant’, late-infantile NCL (vLINCL) [28], [29]. The *CLN6*-encoded protein, linclin or CLN6p, encodes an ER-resident, multipass transmembrane protein. Though CLN6p function has not yet been well studied, it is also implicated in trafficking and regulating lysosomal function [30], [31].

We previously established genetically precise murine and cell-based models for JNCL, *Cln3* ^Δex7/8^ mice and Cb*Cln3* ^Δex7/8^ cerebellar cells [20], [32]. Here, we have created an analogous cellular model from *Cln6* ^nclf^ spontaneous mutant mice, originally identified at The Jackson Laboratories [33], which harbor a mutation in the murine *Cln6* gene that is also found in vLINCL patients [28], [29]. With this set of genetic cell culture reagents, we have performed detailed, comparative phenotyping in order to determine the degree of overlap in abnormal cellular processes resulting from distinct NCL mutations.

## Results

### Cb*Cln3* ^Δex7/8^ and Cb*Cln6* ^nclf^ neuronal precursor cell lines

We previously established Cb*Cln3* ^Δex7/8^ neuronal precursor cell lines by conditionally immortalizing cerebellar granule neurons from postnatal wild-type, heterozygous or homozygous littermate *Cln3* ^Δex7/8^ mice [20]. These mice bear a ∼1 kb genomic deletion in the endogenous murine *Cln3* gene that is analogous to the most common ∼1 kb genomic deletion in juvenile NCL patients. To permit a comparison of the effects of the vLINCL mutation, we have now created wild-type, heterozygous, and homozygous Cb*Cln6* ^nclf^ neuronal precursor cell lines from postnatal *Cln6* ^nclf^ mice [33], which bear a frameshift-producing, single base-pair insertion in the murine *Cln6* gene, which is also found in human vLINCL patients [28], [29]. The generation of the murine vLINCL cell panel is described in detail in Materials and Methods, along with the characterization demonstrating expression of the neural stem cell marker, nestin, confirming a neuronal lineage (Figure S1).

### Homozygous Cb*Cln6* ^nclf^ and Cb*Cln3* ^Δex7/8^ cells accumulate ATP synthase, subunit c

**Figure 1:**
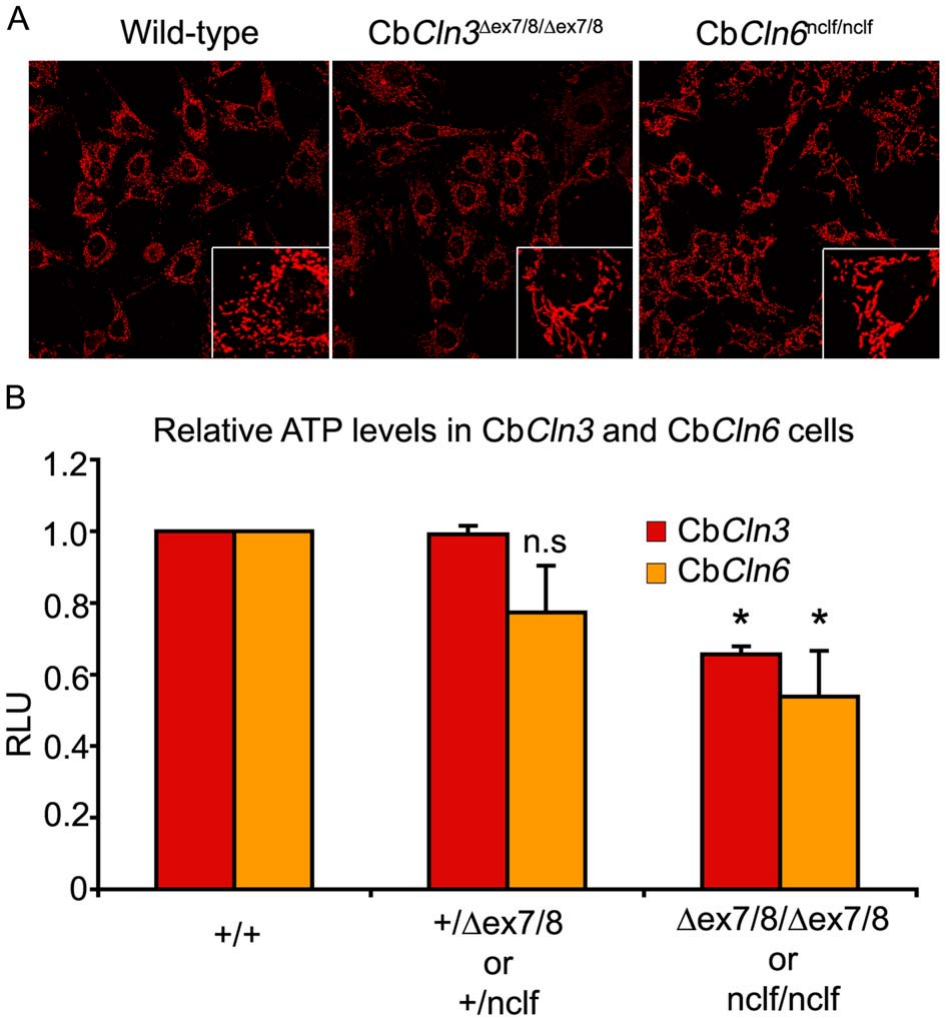
Mitochondrial abnormalities in homozygous Cb*Cln6* ^nclf^ and Cb*Cln3* ^Δex7/8^ cells. **A.** Representative micrographs of wild-type, Cb*Cln3* ^Δex7/8/Δex7/8^ and Cb*Cln6* ^nclf/nclf^ cells immunostained with antibody recognizing the mitochondrial matrix protein, grp75, are shown. Note the elongated appearance of mitochondria in homozygous Cb*Cln3* ^Δex7/8^ and Cb*Cln6* ^nclf^ cells, compared to wild-type cells in the zoomed insets. A lower magnification of a representative field of cells is also shown to demonstrate the subconfluent culture conditions. The altered mitochondrial morphology was also quantified by automated image analysis, showing a significantly reduced circularity index of the labeled mitochondria in the mutant cells, compared to wild-type cells (see Results). Wild-type panel is representative of both Cb*Cln3* ^+/+^ and Cb*Cln6* ^+/+^ cell lines. Heterozygous cell lines were indistinguishable from wild-type (not shown). All images were captured at 40× magnification and were taken on the same day with identical settings. **B.** The bar graph depicts relative total cellular ATP levels in wild-type, heterozygous, or homozygous Cb*Cln3* ^Δex7/8^ and Cb*Cln6* ^nclf^ cells, determined using the CellTiter-GLO® Luminescent Cell assay. Relative luciferase units were normalized to the wild-type cell lines and were pooled from 2–3 independent assays per cell line, each tested in 3–10 wells per assay. For reference, absolute RLUs for Cb*Cln3* ^+/+^ and Cb*Cln6* ^+/+^ cell lines were 1995830±27506 and 626172±151671, respectively. *, p≤.01 in a Student's t-test; n.s. = not significant.

The pathological hallmark of NCL is an abnormal lysosomal accumulation of the pore-forming subunit c of the mitochondrial F_0_ ATP synthase complex. At sub-confluent density, a mitochondrial marker, anti-grp75, which has revealed significant elongation of mitochondria in homozygous Cb*Cln3* ^Δex7/8^ cells [20] (mean circularity index 0.81±.002), also revealed abnormally elongated mitochondria in homozygous Cb*Cln6* ^nclf^ cerebellar cells (mean circularity index 0.79±.003), compared to wild-type cells (Cb*Cln3* ^+/+^ or Cb*Cln6* ^+/+^; mean circularity index 0.85±.002) (Figure 1A). Similar to homozygous Cb*Cln3* ^Δex7/8^ cells, the homozygous Cb*Cln6* ^nclf^ cerebellar cells also displayed a significant reduction (∼30–40%, p≤.01) in total cellular ATP levels, relative to wild-type or heterozygous cells (Figure 1B), strongly suggesting altered mitochondrial morphology and function.

**Figure 2:**
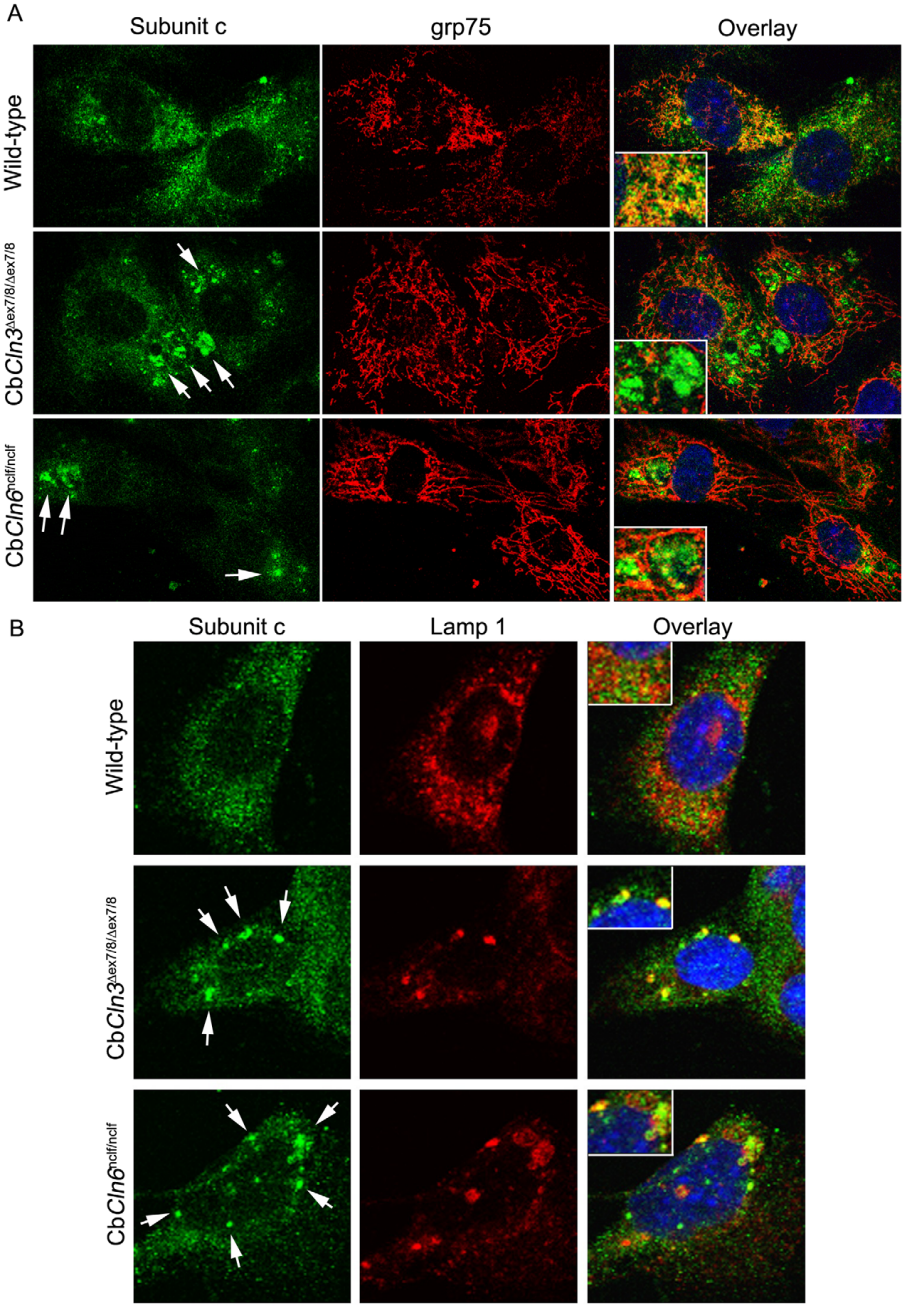
Subunit c deposits co-localize with Lamp 1 in homozygous Cb*Cln3* ^Δex7/8^ and Cb*Cln6* ^nclf^ cells. **A.** Representative micrographs of confluency aged wild-type, Cb*Cln3* ^Δex7/8/Δex7/8^ and Cb*Cln6* ^nclf/nclf^ cells, co-immunostained with antibodies recognizing subunit c (green) and the mitochondrial marker, grp75 (red). Note the large accumulations of subunit c immunostain (white arrows) in both Cb*Cln3* ^Δex7/8/Δex7/8^ and Cb*Cln6* ^nclf/nclf^ cells, but which are not common in the wild-type cells. Moderate overlap of the subunit c and grp75 immunostains is observed in wild-type cells (yellow in overlay), but little to no overlap is seen in Cb*Cln3* ^Δex7/8/Δex7/8^ and Cb*Cln6* ^nclf/nclf^ cells. Also, again note the elongated mitochondrial morphology revealed by the grp75 immunostain in Cb*Cln3* ^Δex7/8/Δex7/8^ and Cb*Cln6* ^nclf/nclf^ cells, compared to wild-type cells. **B.** Representative micrographs of confluency aged wild-type, Cb*Cln3* ^Δex7/8/Δex7/8^, and Cb*Cln6* ^nclf/nclf^ cells, co-immunostained with antibodies recognizing subunit c (green) and Lamp 1 (red). Limited overlap of subunit c and Lamp 1 immunostain is observed in wild-type cells (yellow in overlay), but Lamp 1 strongly, though not perfectly, overlaps with the accumulated subunit c in Cb*Cln3* ^Δex7/8/Δex7/8^ and Cb*Cln6* ^nclf/nclf^ cells. Note that in confluency aged cultures, the Lamp 1-labeled compartment appears expanded and/or aggregated in the mutant cells, which was not observed under sub-confluent culture conditions (not shown). **A,B.** Insets provide a zoomed view of the degree of immunostain overlap (yellow). Blue = DAPI stain. Images were captured with a 40X objective and, for like stains, were taken on the same day with identical settings.

Homozygous Cb*Cln3* ^Δex7/8^ cells, when aged at confluent cell density, accumulated subunit c-positive puncta [20]. Assessment of the wild-type and Cb*Cln6* ^nclf^ cerebellar cells revealed subunit c-positive puncta in aged homozygous mutant Cb*Cln6* ^nclf^ cerebellar cells, similar to that observed in the aged homozygous Cb*Cln3* ^Δex7/8^ cells (Figure 2). Consistent with the abnormal accumulation occurring in a lysosomal compartment, little to no overlap of subunit c signal was observed with mitochondrial grp75 in confluent aged homozygous Cb*Cln3* ^Δex7/8^ cells and only limited overlap of the subunit c and grp75 immunostain was observed in the confluent aged homozygous Cb*Cln6* ^nclf^ cerebellar cells (Figure 2A), while the subunit c immunostain in confluent aged wild-type cells exhibited moderate overlap with grp75 immunostain (Figure 2A). Moreover, in the aged wild-type cerebellar cells, the subunit c immunostain did not overlap with Lamp 1 immunostain, whereas the strongly immunopositive subunit c puncta in both the homozygous Cb*Cln3* ^Δex7/8^ and Cb*Cln6* ^nclf^ cerebellar cells aged at confluent density were Lamp 1-positive (Figure 2B), though the overlap was imperfect, particularly in the homozygous Cb*Cln6* ^nclf^ cerebellar cells. In both homozygous Cb*Cln3* ^Δex7/8^ and Cb*Cln6* ^nclf^ cells aged at confluent density, the Lamp 1 immunostain also revealed enlarged or aggregated lysosomes, which were not often observed in the aged wild-type cells, consistent with lysosomal defects.

Thus, cerebellar cells homozygous for either a vLINCL mutation or the major JNCL mutation exhibited aberrant mitochondrial morphology and, when aged at confluent density, accumulated mitochondrial ATP synthase subunit c in enlarged, Lamp 1-positive vesicles, albeit with slightly different staining characteristics.

### Homozygous Cb*Cln6* ^nclf^ and Cb*Cln3* ^Δex7/8^ cells display similar but distinct membrane abnormalities

**Figure 3:**
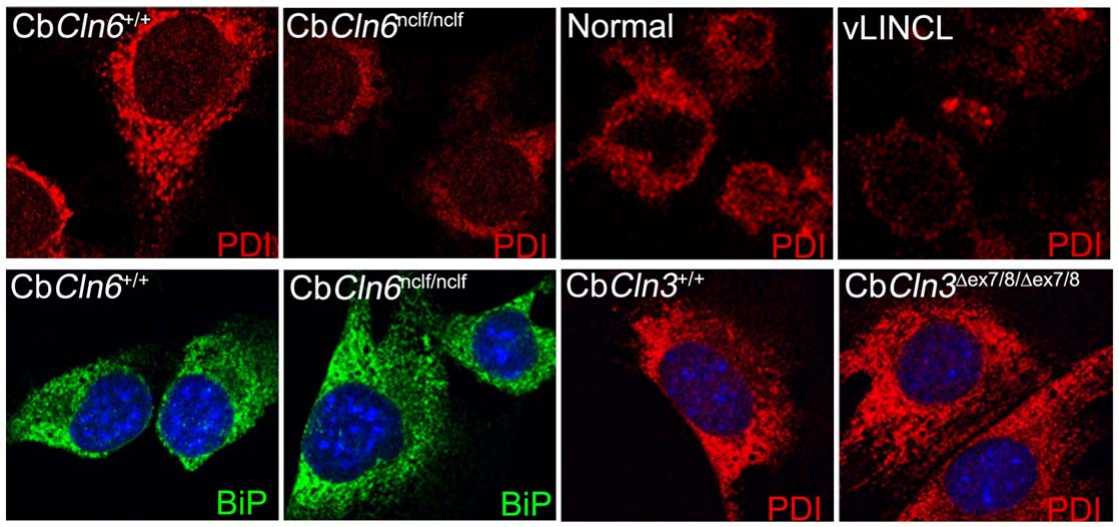
Altered PDI immunostain in homozygous Cb*Cln6* ^nclf^ cells. Representative micrographs of Cb*Cln6* (Cb*Cln6* ^+/+^ and Cb*Cln6* ^nclf/nclf^) and Cb*Cln3* cells (Cb*Cln3* ^Δex7/8/Δex7/8^; Cb*Cln3* ^+/+^) immunostained for the ER marker protein PDI (red) are shown. Note the decreased PDI signal in Cb*Cln6* ^nclf/nclf^ cells, as compared to wild-type (Cb*Cln6* ^+/+^ or Cb*Cln3* ^+/+^) and Cb*Cln3* ^Δex7/8/Δex7/8^ cells. PDI immunostain of normal and variant late-infantile (vLINCL) lymphoblast cells is also shown. BiP immunostain was comparable across all of the cerebellar cell lines, but is only shown for the Cb*Cln6* ^+/+^ and Cb*Cln6* ^nclf/nclf^ cells. BiP was not assessed in lymphoblast cells. All images were taken at 40× magnification, and for like stains, on the same day, with identical instrument settings. DAPI nuclear counter stain is shown in blue.

To determine whether similar accumulation of subunit c in Lamp 1-positive vesicles, might reflect similar or distinct membrane organelle perturbations, Cb*Cln6* ^nclf/nclf^ and Cb*Cln3* ^Δex7/8/Δex7/8^ cerebellar cells were assessed using a panel of organelle markers [20]. We found no obvious morphological differences in the *cis*- and *trans*-Golgi in either homozygous Cb*Cln3* ^Δex7/8^ or Cb*Cln6* ^nclf^ cerebellar cells (data not shown). However, the ER marker protein, PDI, consistently displayed reduced staining intensity in homozygous Cb*Cln6* ^nclf^ cerebellar cells, compared to wild-type (Cb*Cln6* ^+/+^) cells (Figure 3, top panels). Homozygous Cb*Cln3* ^Δex7/8^ cells did not display altered PDI immunostain (Figure 3). Interestingly, immunostain for another ER-associated protein, BiP [34], was not decreased in homozygous Cb*Cln6* ^nclf^ cerebellar cells and highlighted a morphologically intact ER network (Figure 3). Notably, PDI immunostain was also decreased in variant late-infantile NCL patient lymphoblast cells, compared to normal lymphoblasts (Figure 3, bottom panels), suggesting that the vLINCL mutation may specifically alter PDI epitope availability or distribution within the ER of diverse cell types. Total PDI levels were not obviously different in the homozygous Cb*Cln6* ^nclf^ cerebellar cells versus wild-type cells by immunoblot analysis (data not shown).

**Figure 4:**
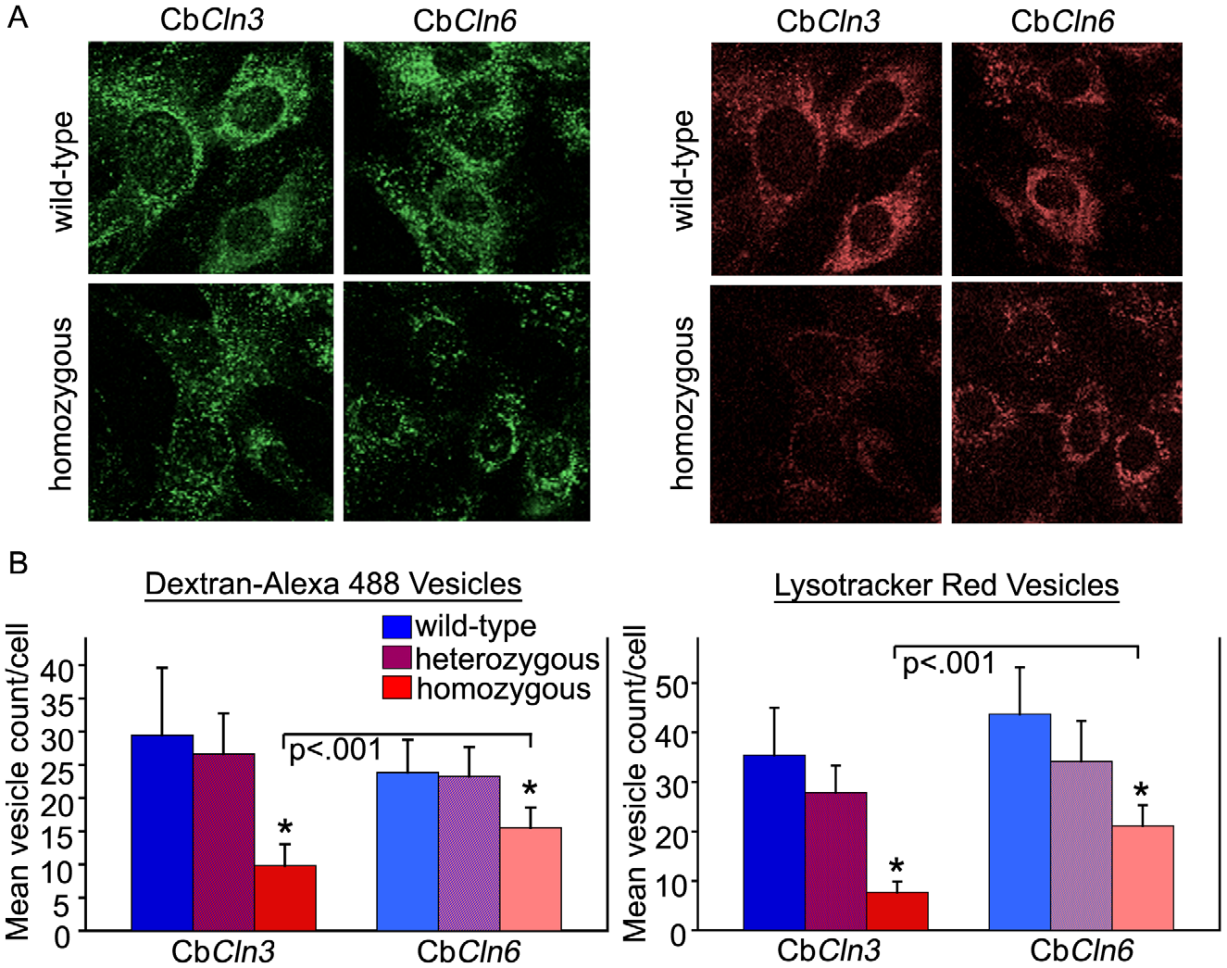
Altered fluid-phase endocytosis and LysoTracker® stain in homozygous Cb*Cln3* ^Δex7/8^ and Cb*Cln6* ^nclf^ cells. **A.** Representative micrographs of Cb*Cln3* cells (wild-type and homozygous) and Cb*Cln6* (wild-type and homozygous) cells stained with the fluid-phase endocytic marker, dextran Alexa-488 (green), and the lysosomal marker, LysoTracker®-Red (red), are shown. Reduced vesicular staining is evident in the homozygous cells, compared to wild-type cells for both Cb*Cln3* and Cb*Cln6* cells. However, note the differential distribution pattern of the labeled vesicles in homozygous Cb*Cln3* ^Δex7/8^ cells, compared to homozygous Cb*Cln6* ^nclf^ cells. Labeled vesicles in homozygous Cb*Cln6* ^nclf^ cells remain perinuclear localized, as they appear in wild-type cell lines. To the contrary, labeled vesicles in homozygous Cb*Cln3* ^Δex7/8^ cells appear less perinuclear localized, compared to wild-type cells and homozygous Cb*Cln6* ^nclf^ cells. All images were captured at 40× magnification and were taken on the same day with identical settings. **B.** Bar graphs depicting quantification of labeled endosomes (left) and lysosomes (right) in Cb*Cln3* cells (Cb*Cln3* ^+/+^ and Cb*Cln3* ^Δex7/8/Δex7/8^) and Cb*Cln6* (Cb*Cln6* ^+/+^ and Cb*Cln6* ^nclf/nclf^) cells are shown. Mean vesicle count/cell was significantly reduced (*p<.001) in both homozygous Cb*Cln3* ^Δex7/8^and Cb*Cln6* ^nclf^ cells, compared to the respective wild-type or heterozygous cell lines. A student's t-test also revealed a significant difference (p<.001) between the homozygous cell lines of differing genotype (i.e. Cb*Cln3* ^Δex7/8/Δex7/8^ versus Cb*Cln6* ^nclf/nclf^), which was suggestive of a more dramatic reduction of stained vesicles in the Cb*Cln3* ^Δex7/8/Δex7/8^ cells.

To determine whether subconfluent, non-stressed Cb*Cln6* ^nclf^ cerebellar neuronal precursor cells displayed abnormalities in the endosomal-lysosomal system, which is significantly disrupted in subconfluent homozygous Cb*Cln3* ^Δex7/8^ cells [20], we assayed fluid phase endocytosis using a fluorescently labeled dextran uptake assay (dextran, Alexa Fluor® 488) and acidic organelles were probed using LysoTracker® Red. As expected, homozygous Cb*Cln3* ^Δex7/8^ cells showed consistently reduced numbers of dextran-Alexa 488 labeled vesicles and LysoTracker® stained vesicles, along with a reduced perinuclear distribution of the labeled vesicles, compared to wild-type (Cb*Cln3* ^+/+^) or heterozygous (Cb*Cln3* ^+/Δex7/8^) cells (Figure 4). Homozygous Cb*Cln6* ^nclf^ cells also showed consistently reduced dextran-Alexa 488 labeled vesicles and LysoTracker® stained vesicles, compared to wild-type (Cb*Cln6* ^+/+^) or heterozygous (Cb*Cln6* ^+/nclf^) cells. However, in contrast to what was observed in homozygous Cb*Cln3* ^Δex7/8^ cells, the distribution of the labeled vesicles in homozygous Cb*Cln6* ^nclf^ cells was not obviously altered (Figure 4A). Notably, the expanded and/or aggregated lysosomal compartment, which was evident by Lamp 1 immunostain in the confluent density aged homozygous Cb*Cln3* ^Δex7/8^ and Cb*Cln6* ^nclf^ cells, as shown in Figure 2B, was not observed in the subconfluent, Lysotracker-stained homozygous Cb*Cln3* ^Δex7/8^ and Cb*Cln6* ^nclf^ cells here. Automated image analysis demonstrated that the numbers of labeled vesicles were more dramatically decreased (p<.001) in the homozygous Cb*Cln3* ^Δex7/8^ cells (∼65–75% reduced from wild-type levels), than in the homozygous Cb*Cln6* ^nclf^ cells (∼35–50% reduced from wild-type levels) (Figure 4B).

### Homozygous Cb*Cln*3^Δex7/8^ and Cb*Cln6* ^nclf^ cerebellar cells exhibit mostly distinct gene expression changes

The membrane organelle survey revealed similarities but also differences in the impact of the vLINCL and JNCL mutations that implied distinct underlying processes might be involved. To assess this idea at the molecular level, we performed unbiased global gene expression analyses on total RNA isolated from wild-type (Cb*Cln3* ^+/+^ and Cb*Cln6* ^+/+^) and homozygous Cb*Cln3* ^Δex7/8^ and Cb*Cln6* ^nclf^ cerebellar cells (see Materials and Methods).

**Figure 5:**
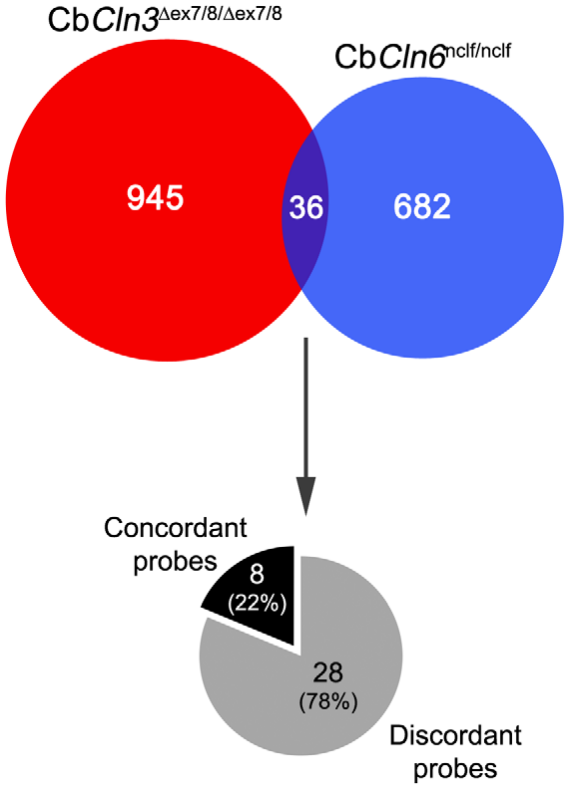
Venn diagram depicting degree of overlap in gene expression changes in homozygous Cb*Cln3* ^Δex7/8^ and Cb*Cln6* ^nclf^ cells. 981 probes were significantly changed in Cb*Cln3* ^Δex7/8/Δex7/8^ cells, compared to Cb*Cln3* ^+/+^ cells (red). 718 probes were significantly changed in Cb*Cln6* ^nclf/nclf^ cells, compared to Cb*Cln6* ^+/+^ cells (blue). A ±1.4-fold change and p≤0.01 cut-off was applied to generate these datasets. As represented by the pie chart, among the 36 shared probes, 8 (22%) were concordant in their direction of change in the Cb*Cln3* ^Δex7/8/Δex7/8^ and Cb*Cln6* ^nclf/nclf^ cells, while 28 (78%) were discordant in their direction of change in the Cb*Cln3* ^Δex7/8/Δex7/8^ and Cb*Cln6* ^nclf/nclf^ cells.

In a probe-level analysis, we identified 981 significantly changed probes in the homozygous Cb*Cln3* ^Δex7/8^ cerebellar cells (p<.01, absolute fold-change>1.5; Figure 5, Table S1) and 718 significantly changed probes in the homozygous Cb*Cln6* ^nclf^ cerebellar cells (p<.01, absolute fold-change>1.5; Figure 5, Table S2). Among these, only a small number, 36 probes, were significantly changed in both homozygous Cb*Cln3* ^Δex7/8^ and Cb*Cln6* ^nclf^ cells, with the majority (28) discordant in the direction of change (Figure 5, Table 1). The reliability of the datasets was assessed by principal components analysis (PCA) (Figure S2) and real-time qRT-PCR of selected genes (Table S3).

**Table 1:** Significant probes shared between Cb*Cln6* ^nclf^ and Cb*Cln3* ^Δex7/8^datasets.

Probe	Gene Symbol	Cb*Cln6* ^nclf/nclf^ Cells Fold Change	Cb*Cln3* ^Δex7/8/Δex7/8^ Cells Fold Change
1417505_s_at	Il11ra1	−43.6	30.3
1418675_at	Osmr	−23.6	20.6
1459888_at	LOC545261	−14.5	13.5
1447975_at	LOC545261	−5.7	5.0
1433653_at	BC029169	−11.3	8.9
1437760_at	Galnt12	−7.3	12.7
1416516_at	Fscn1	−4.7*	−1.9*
1448378_at	Fscn1	−2.9*	−2.0*
1448901_at	Cpxm1	−3.2	5.1
1455604_at	Fzd5	−3.1	−2.6
1419664_at	Srr	−2.7	2.7
1435830_a_at	5430435G22Rik	−2.4	2.7
1426306_a_at	Maged2	−2.3	4.4
1436033_at	BC031353	−2.1	3.3
1452034_at	Prepl	−1.8	−1.7
1432526_a_at	Snf8	−1.8*	1.6*
1433668_at	Pnrc1	−1.7	2.1
1448343_a_at	Nbr1	−1.7	2.0
1436795_at	9630058J23Rik	−1.6	−1.6
1415776_at	Aldh3a2	−1.6	1.6
1452094_at	P4ha1	−1.5	−1.5
1421139_a_at	Zfp386	1.5	−1.7
1434724_at	Usp31	1.6	−2.6
1416004_at	Ywhah	1.7	−1.6
1423682_a_at	Cdca4	1.9	−1.8
1417024_at	Hars	2.0	−1.6
1435467_at	Fgd6	2.0	−2.4
1424675_at	Slc39a6	2.2	−1.6
1455321_at	Ddhd1	2.2	2.2
1421103_at	Bmp2k	2.2	−7.0
1455829_at	Usp14	2.4	−1.6
1416208_at	Usp14	2.4	−1.5
1451818_at	Mib1	2.7	−1.6
1423735_a_at	Wdr36	2.7	−1.5
1459885_s_at	Cox7c	3.6	−2.9
1457632_s_at	Meis2	4.0	1.6

*Validated by qRT-PCR.

Gene ontology analysis of the gene lists did not reveal obvious functional overlap among the genes with altered expression in both Cb*Cln3* ^Δex7/8/Δex7/8^ and Cb*Cln6* ^nclf/nclf^ cells, suggesting the pathways most dramatically affected by the *Cln3* ^Δex7/8^ and *Cln6* ^nclf^ mutations were mostly different (Tables S4 and S5). PCA analysis of the Cb*Cln3* ^Δex7/8^ significant probes in the Cb*Cln6* ^nclf^ dataset, and vice versa, further supported this conclusion (Figure S3).

Notably, in our Affymetrix gene expression array analysis, *Cln3* and *Cln6* gene expression were not significantly altered by the vLINCL and JNCL mutations, respectively (Tables S1 and S2). However, subsequent qRT-PCR analysis revealed reduced expression of mutant *Cln3* mRNA in Cb*Cln3* ^Δex7/8/Δex7/8^ cerebellar cells (5-fold downregulated), consistent with previous reports [20], [32], and reduced expression of mutant *Cln6* mRNA in Cb*Cln6* ^nclf/nclf^ cerebellar cells (6-fold downregulated). We also detected a 1.6-fold upregulation of *Cln6* mRNA in homozygous Cb*Cln3* ^Δex7/8^ cells and an ∼2-fold downregulation of *Cln3* mRNA in homozygous Cb*Cln6* ^nclf^ cerebellar cells by qRT-PCR (Table S3). This apparent discrepancy between the Affymetrix array and qRT-PCR data at the level of the *Cln3* and *Cln6* genes could be due to the gene regions being probed in the two different formats, which differed (e.g. reflecting detection of different splice variants), or the different sensitivities of the two methodologies. Among the other NCL loci represented on the Affymetrix array (*Ppt1, Tpp1, Cln5, Mfsd8, Ctsd, Cln8*), only *Cln5*, which is mutated in another form of non-classical ‘variant’ LINCL [35], displayed a significant change (1.8-fold upregulated), and only in the homozygous Cb*Cln3* ^Δex7/8^ cells (Table S1).

Though our probe-level analysis suggested only limited overlap in the genes affected by the *Cln3* ^Δex7/8^ and *Cln6* ^nclf^ mutation, we also sought to examine the data at a pathways level. Gene Set Enrichment Analysis (GSEA) has proven to be a sensitive method of identifying pathways relevant to human disease from gene expression datasets (e.g. [36], [37], [38], [39]). GSEA is predicated on the idea that genes with related biological functions are oftentimes coordinately regulated, and that even small gene expression changes, which escape identification through traditional single gene analysis approaches, are biologically meaningful if in the context of related gene changes [36], [40]. We further analyzed our unbiased gene expression data in the sigPathway program, which is an extension of the original GSEA platform [41], using our entire Cb*Cln3* ^Δex7/8^ and Cb*Cln6* ^nclf^ datasets.

The significantly altered gene sets (false discovery rate<0.01) in homozygous Cb*Cln3* ^Δex7/8^ and Cb*Cln6* ^nclf^ cells are shown in Tables S6 and S7, respectively. Overall, more changed gene sets (597 out of 2077 total screened) were identified in the homozygous Cb*Cln6* ^nclf^ cerebellar cells than in the homozygous Cb*Cln3* ^Δex7/8^ cells (222 out of 2077 total screened). Consistent with our probe-level analysis results, inspection of the top 20 ranked (NTk Rank) gene sets, which reflected those gene sets determined by the sigPathway software to have the most changes among all gene sets screened (see Materials and Methods and [41]), revealed limited overlap in the most dramatically changed pathways in the Cb*Cln3* ^Δex7/8/Δex7/8^ and Cb*Cln6* ^nclf/nclf^ cells (Tables 2 and 3). Only the gene set for the oxidative phosphorylation KEGG pathway scored within the top 20 ranks for both the homozygous Cb*Cln3* ^Δex7/8^ (NTk Rank = 1) and Cb*Cln6* ^nclf^ (NTk Rank = 17) gene set lists. However, supporting the notion that there is convergence in the biological processes affected by the JNCL and vLINCL mutations, further inspection of the complete lists of significantly changed gene sets in the two different genetic cell models revealed a significant negative correlation between them, reflecting common pathways that were changed in the opposite direction in Cb*Cln6* ^nclf/nclf^ and Cb*Cln3* ^Δex7/8/Δex7/8^ cells (Figure S4).

**Table 2:** 20 highest-ranked gene sets significantly altered in Cb*Cln3* ^Δex7/8/Δex7/8^ cerebellar cells.

	Cb*Cln3* ^Δex7/8Δex7/8^ Cells Dataset		Cb*Cln6* ^nclf/nclf^ Cells Dataset	
Pathway	NTk Rank*	NTk Stat	NTk Rank	NTk Stat
KEGG:Oxidative_phosphorylation	1.0	12.3	17.3	−10.2
GO:0015078 hydrogen ion transporter activity	2.0	11.4	23.0	−8.9
GO:0015077 monovalent inorganic cation transporter activity	3.3	11.0	24.3	−8.8
KEGG:Fatty_acid_metabolism	4.0	10.9	354.2	−2.9
GO:0003954; GO:0050136; GO:0008137; GO:0015081 NADH dehydrogenase activity	5.3	10.9	192.7	−3.5
KEGG:Porphyrin_and_chlorophyll_metabolism	5.3	10.8	271.5	−3.1
GO:0016655 oxidoreductase activity, acting on NADH or NADPH, quinone or similar compound as acceptor	7.0	10.6	149.2	−4.7
KEGG:Valine,_leucine_and_isoleucine_degradation	8.0	10.1	427.8	−2.8
GO:0015399 primary active transporter activity	9.0	10.1	32.0	−8.2
GO:0016667 oxidoreductase activity, acting on sulfur group of donors	10.3	−9.7	892.2	−1.9
GO:0015036 disulfide oxidoreductase activity	10.7	−9.6	1268.0	−1.1
KEGG:Ribosome	12.0	8.9	27.0	−8.7
GO:0006119 oxidative phosphorylation	13.0	8.8	182.0	−3.6
GO:0046873 metal ion transporter activity	14.0	8.7	86.5	−6.0
GO:0042773 ATP synthesis coupled electron transport	15.0	8.4	246.2	−3.2
GO:0005773 vacuole	16.0	8.0	172.2	−3.7
GO:0000323; lytic vacuole GO:0005764; lysosome	17.7	7.9	220.8	−3.3
GO:0016491 oxidoreductase activity	18.0	7.8	52.0	−6.8
GO:0019370 leukotriene biosynthesis	18.3	−7.8	1220.3	1.2
GO:0006691 leukotriene metabolism	21.3	−7.5	1157.3	1.4

*The top 20 NTk ranked gene set pathways in the Cb*Cln3* ^Δex7/8^ dataset are shown (NTk Rank), ranked by the sigPathway program according to the average NTk statistics (NTk Stat). NTk statistics reflect how significant changes were within a given gene set (the higher the absolute value of the statistic, the greater its significance), and the direction of change (indicated by positive or negative statistics). For comparison, the Cb*Cln6* ^nclf^ dataset NTk Rank and NTk Stat values are also shown. Replicated gene sets were grouped together. For example, four different gene sets, categorized ‘NADH dehydrogenase activity’, gave identical statistics, so they are grouped together into one row of the table, with the individual gene set identifiers listed in the ‘Pathway’ column.

**Table 3:** 20 highest-ranked gene sets significantly altered in Cb*Cln6* ^nclf/nclf^ cerebellar cells.

	Cb*Cln6* ^nclf/nclf^ Cells Dataset		Cb*Cln3* ^Δex7/8/Δex7/8^ Cells Dataset	
Pathway	NTk Rank*	NTk Stat	NTk Rank	NTk Stat
GO:0001584 rhodopsin-like receptor activity	1.0	12.5	1044.0	−1.1
GO:0004930 G-protein coupled receptor activity	2.0	12.3	1474.7	−0.6
GO:0005783 endoplasmic reticulum	3.0	−12.1	46.7	5.0
GO:0009059 macromolecule biosynthesis	4.0	−11.8	732.2	1.6
GO:0006412 protein biosynthesis	5.0	−11.6	846.5	1.4
GO:0004888 transmembrane receptor activity	6.0	11.5	423.5	2.4
GO:0015031 protein transport	7.3	−11.1	53.0	4.5
KEGG: Neuroactive_ligand-receptor_interaction	8.0	11.1	612.2	−1.9
GO:0045184 establishment of protein localization	9.3	−11.0	54.7	4.5
GO:0015268 alpha-type channel activity	10.3	10.9	1624.0	−0.4
GO:0008104 protein localization	11.0	−10.8	51.3	4.6
GO:0031090 organelle membrane	11.0	−10.9	34.0	6.0
GO:0015267 channel or pore class transporter activity	13.3	10.6	839.5	−1.4
GO:0007186 G-protein coupled receptor protein signaling pathway	13.7	10.6	413.0	−2.4
GO:0005216 ion channel activity	15.0	10.4	1395.0	−0.6
GO:0005840 ribosome	16.0	−10.3	26.3	6.9
KEGG: Oxidative_phosphorylation	17.3	−10.2	1.0	12.3
GO:0003735 structural constituent of ribosome	17.7	−10.2	24.0	7.0
GO:0030529 ribonucleoprotein complex	19.0	−9.8	442.5	2.4
GO:0042165 neurotransmitter binding	20.0	9.6	768.3	−1.6

*The top 20 NTk ranked gene set pathways in the Cb*Cln6* ^nclf/nclf^ dataset are shown (NTk Rank), ranked by the sigPathway program according to the average NTk statistics (NTk Stat). NTk statistics reflect how significant changes were within a given gene set (the higher the absolute value of the statistic, the greater its significance), and the direction of change (indicated by positive or negative statistics). For comparison, the Cb*Cln3* ^Δex7/8^ dataset NTk Rank and NTk Stat values are also shown.

Intriguingly, consistent with biological data supporting a lysosomal localization and function for the *Cln3*-encoded protein, lysosomal function-related (GO:0005773, GO:0000323, GO:0005764) gene sets were among the top 20 in the homozygous Cb*Cln3* ^Δex7/8^ list, but not in the homozygous Cb*Cln6* ^nclf^ gene set list (Table 2). Conversely, consistent with the ER localization of the *Cln6*-encoded protein, ER-function (GO:0005783), protein biosynthesis (GO:0009059, GO:0006412) and protein transport-related (GO:0015031, GO:0045184, GO:0008104) gene sets were among the top 20 in the homozygous Cb*Cln6* ^nclf^ list (Table 3), but were ranked substantially lower in the homozygous Cb*Cln3* ^Δex7/8^ gene set list.

Thus, our unbiased global gene expression analysis of Cb*Cln3* ^Δex7/8^ and Cb*Cln6* ^nclf^ cerebellar cells lends further support for the hypothesis that *Cln3* and *Cln6* mutations initiate disease via distinct molecular and cell biological processes that converge on a common pathway. Moreover, a number of potentially relevant pathways have been identified, including oxidative phosphorylation, that merit further investigation into their role in the NCL disease process.

## Discussion

The NCLs, while genetically heterogeneous, share a common pathological feature, the accumulation and storage of ceroid lipofuscin, which appears to mostly be comprised of dolichol lipids and the hydrophobic protein, subunit c of mitochochondrial ATP synthase [4], [5], [42]. Here, we have shown that homozygous Cb*Cln6* ^nclf^ cerebellar cells, like homozygous Cb*Cln3* ^Δex7/8^ cerebellar cells, can be induced to accumulate mitochondrial ATP synthase subunit c in Lamp-1 positive compartments by aging in confluent culture conditions. The results of our comparative analyses in sub-confluent cultured homozygous Cb*Cln6* ^nclf^ and Cb*Cln3* ^Δex7/8^ cerebellar cells have distinguished the underlying biological and molecular processes that presage this shared overt disease-associated storage of subunit c. Moreover, the divergent early processes uncovered here strongly support the hypothesis that the *CLN6* and *CLN3*-encoded proteins serve distinct functions in neuronal cells.

Indeed, consistent with the predominant ER localization of CLN6p, the vLINCL mutation appears to significantly impact the ER, strongly implicating CLN6p in the proper function of this important membrane organelle. For example, PDI immunostain was specifically decreased in homozygous Cb*Cln6* ^nclf^ cells and patient lymphoblasts, suggesting altered disposition of this protein-folding chaperone, and the expression of ER-related gene sets were among the highest ranked in homozygous Cb*Cln6* ^nclf^ cells, suggesting that CLN6p is required for ER function and protein biosynthesis, perhaps of endosomal-lysosomal proteins given the clear impact of the loss of CLN6p function on endocytosis and lysosomal turnover of the subunit c protein. Why PDI immunostain was altered in the homozygous Cb*Cln6* ^nclf^ cells remains unclear, but the lack of a concomitant significant change in BiP suggested that these cells were not undergoing a global unfolded protein response. Further study of the PDI phenotype is needed to shed new light on the specific ER-associated pathways connected to CLN6p dysfunction. It was also intriguing that our pathways analysis here specifically highlighted gene expression changes in membrane-associated receptor proteins in the homozygous Cb*Cln6* ^nclf^ cells, which is suggestive of a role for CLN6p in the regulation of this protein family.

Similarly, consistent with the primary localization of CLN3p within the endosomal-lysosomal system, ER biology was not dramatically altered by the JNCL mutation but instead the results of our pathways analysis implied that lysosomal biology was strongly affected in the homozygous Cb*Cln3* ^Δex7/8^ cerebellar cells, which was consistent with biological data in this study and others (reviewed in [9]). Moreover, relatively specific changes in gene sets related to metabolic processes, such as fatty acid and amino acid metabolism, and in ion transport in Cb*Cln3* ^Δex7/8^ cerebellar cells, which were not evidently dramatically changed in homozygous Cb*Cln6* ^nclf^ cells, suggests focusing studies aimed at CLN3p function on these processes.

It is noteworthy that homozygous mutation of *Cln3* or *Cln6* in our cerebellar cell models of JNCL and vLINCL did not dramatically alter expression at the other NCL loci, at least by Affymetrix array analysis, supporting distinct primary functions for the differing NCL related proteins, consistent with the observation that NCL patients have varied storage material ultrastructure, age-at-onset, and order of symptom onset that typically correlates with the genetic etiology [13], [14]. However, subtler gene expression changes in the other NCL loci were detected, in particular, in follow-up analysis by the more sensitive method of qRT-PCR, supporting the hypothesis that the NCL gene functions converge on a common pathway.

Biological areas that merit further investigation, because they were commonly altered in cerebellar cells in response to CLN6p and CLN3p dysfunction, are aspects of vesicle/membrane trafficking, protein transport, and altered metabolism. For example, future study of alterations in *Snf8* and *Fscn1*, which were two of the validated gene changes in homozygous Cb*Cln6* ^nclf^ and Cb*Cln3* ^Δex7/8^ cells (Table 1, Table S3), could lead to an improved understanding of the altered trafficking in these two forms of NCL. *Snf8* (a.k.a. ESCRT-II complex subunit VPS22), which is involved in sorting endocytosed and ubiquitinated proteins into multivesicular bodies for subsequent lysosomal delivery and degradation [43], was significantly upregulated in homozygous Cb*Cln3* ^Δex7/8^ cells, and significantly downregulated in homozygous Cb*Cln6* ^nclf^ cells (Tables 1 and S3). *Fscn1* (fascin-1), an actin-bundling protein highly expressed in brain (reviewed in [44]), was downregulated in both homozygous Cb*Cln3* ^Δex7/8^ and homozygous Cb*Cln6* ^nclf^ cells (Tables 1 and S3). The importance of the actin cytoskeleton in membrane trafficking is well documented (reviewed in [45]), and a role for CLN3p in membrane-cytoskeletal interactions has already been proposed [46], [47].

It is reasonable to postulate that the commonly altered molecular genes and pathways in homozygous Cb*Cln6* ^nclf^ and Cb*Cln3* ^Δex7/8^ cells may culminate in the abnormal accumulation of mitochondrial ATP synthase, subunit c protein that becomes manifest when homozygous Cb*Cln6* ^nclf^ and Cb*Cln3* ^Δex7/8^ cells are stressed by aging at confluent cell density. Our co-staining analyses of the formed deposits was suggestive that in both homozygous Cb*Cln6* ^nclf^ and Cb*Cln3* ^Δex7/8^ cells, the accumulation of the subunit c protein occurs within acidic organelles rather than in the mitochondrion itself, consistent with a defect in the autophagosomal-lysosomal pathway in these forms of NCL [22], [48], [49]. The subunit c-positive foci that formed in the homozygous Cb*Cln6* ^nclf^ and Cb*Cln3* ^Δex7/8^ cells were morphologically heterogeneous, so, while dramatic differences were not apparent, it would also be worthwhile to determine whether the vLINCL and JNCL mutations may give rise to subtle differences in the relative kinetics and features of subunit c turnover.

In summary, our data are consistent with distinct functions for the CLN3p and CLN6p proteins, that likely primarily work in distinct processes regulating shared downstream biological pathways that are connected to the lysosome, the mitochondrion and subunit c turnover, in a manner that is essential for proper neuronal cell survival. Our findings, therefore, support the interconnected goals of developing therapeutics based on a full understanding of CLN3p and CLN6p functions, which may prove to be disease-specific, as well as therapeutics aimed at circumventing or preventing the changes proximal to the overall dysfunction of cellular energetics and membrane function that result in storage of ceroid lipofuscin and neuronal cell death.

## Materials and Methods

### Ethics Statement

All mouse protocols were in accordance with the National Institutes of Health Guide for the Care and Use of Laboratory Animals and were reviewed and approved by the Massachusetts General Hospital (MGH) Subcommittee of Research Animal Care (SRAC), which serves as the Institutional Animal Care and Use Committee (IACUC) for MGH (Protocol #2005N000289).

The use of anonymous, de-identified human patient lymphoblast cell lines for our NCL research was reviewed by the Partners Institutional Review Board and deemed exempt (2010P-001489).

### Animals

*Cln6* ^nclf^ spontaneous mutant mice were originally purchased from The Jackson Laboratory, and were subsequently maintained as a breeding colony on the C57Bl6/J background, at Massachusetts General Hospital. Genotypes were determined by the mouse nclf exon 4 insertion assay, from tail biopsy DNA, as previously described [28]. *Cln3* ^Δex7/8^ mutant mice were previously described [32].

### Antibodies and Cell Staining Reagents

Commercial antibodies used were anti-nestin (Rat 401, Developmental Studies Hybridoma Bank, maintained by The University of Iowa, Department of Biological Sciences), anti-GFAP (cat#Z0334, Dako Corporation), anti-PDI (H-160, cat#sc-20132, Santa Cruz Biotechnology), anti-GM130 (cat#G65120, BD Transduction Laboratories), anti-tubulin (Sigma), anti-BiP (Abcam), anti-EEA1 (C-15, cat#sc-6414, Santa Cruz Biotechnology), anti-Rab7 (C-19, cat#sc-6563, Santa Cruz Biotechnology), anti-Lamp 1 (1D4B, cat#sc-19992, Santa Cruz Biotechnology), anti-Grp75 (cat#SPS-825, Stressgen). All fluorescent secondary antibodies used were obtained from Molecular Probes/Invitrogen and were used at a 1∶200 dilution. Cell staining reagents used were LysoTracker® Red DND-99 (500 nM; cat#L-7528, Invitrogen) and 10,000 MW dextran-Alexa Fluor® 488 (1 mg/ml; cat#D-22910, Invitrogen).

A new subunit c antibody was generated to replace the diminishing stocks of the previously described subunit c antibody generated by Dr. Kominami [20], [32], [50]. A peptide corresponding to amino acids 62–73 of the subunit c protein (the N-terminus of the mature protein) was synthesized with an additional C-terminal cysteine residue for carrier protein conjugation by the MGH Peptide Core Facility. Keyhole limpet hemocyanin (KLH)-conjugated peptide was used for rabbit immunization, and antisera were collected and affinity purified according to standard procedures (Quality Controlled Biochemicals). The new anti-subunit c antibody was tested alongside the previously described subunit c antibody [20], [32], [50] and was found to perform similarly in all immunostaining and immunoblot assays (data not shown). All subunit c data contained herein were obtained with the new subunit c antibody, used at 1∶200–1∶500 dilution.

### Generation and maintenance of CbCln6^nclf^ and CbCln3^Δex7/8^ cerebellar neuronal precursor cell lines

Cb*Cln6* ^nclf^ cerebellar neuronal precursor cell lines were established in the same manner as Cb*Cln3* ^Δex7/8^ cerebellar neuronal precursor cells, which were previously described [20]. Briefly, postnatal day 4 (P4) cerebella were dissected from wild-type, heterozygous, and homozygous *Cln6* ^nclf^ (or *Cln3* ^Δex7/8^) littermate mice, and primary cultures were established that were enriched for cerebellar granule neurons, according to previously established procedures [51]. Cultures were then transduced with a retroviral vector containing the tsA58/U19 temperature-sensitive SV40 large T antigen and a neomycin selection marker [52]. Clones were selected for growth in 400 µg/ml G418, and multiple clonal cell lines were isolated for each genotype that expressed the neural stem cell marker, nestin, but lacked glial fibrillary acidic protein (GFAP) expression by immunofluorescence and, in some cases, also confirmed by Western blot, to verify a neuronal lineage (Figure S1 and [20]). Once established, cell lines were re-genotyped using previously described *Cln6* ^nclf^ and *Cln3* ^Δex7/8^ genotyping assays [20], [28]. The expression and endoplasmic reticular-localization of the CLN6p protein was confirmed in the wild-type cells, and significantly reduced expression was observed in the homozygous Cb*Cln6* ^nclf^ cells, consistent with previous reports [31], [53] (data not shown).

For maintenance, Cb*Cln6* ^nclf^ and Cb*Cln3* ^Δex7/8^ cerebellar neuronal precursor cells were grown between 30 and 90% confluency on plastic tissue culture dishes in ‘Cbc’ media (Dulbecco's Modified Eagle Medium [DMEM; Gibco BRL #11995-065], 10% heat-inactivated fetal bovine serum [FBS; Sigma #12306C], 24 mM KCl, penicillin/streptomycin/glutamine [1X, Gibco BRL], and G418 [200 µg/ml] to maintain selection), in a water-jacketed, humidified incubator maintained at 33°C, 5% CO_2_ atmosphere. Passage number was recorded and cells were used for experiments up to ∼passage 15, without apparent impact on phenotypes. All phenotypes were tested in 2–3 independent cell lines per genotype to ensure they represented a genotype-phenotype relationship, rather than just inter-subclone variability. Except where noted, data shown were from representative cell lines. Mycoplasma testing was routinely carried out using the MycoAlert Mycoplasma Detection Kit (Lonza #LT07-118), according to the manufacturer's recommendations, to ensure no mycoplasma contamination of cell culture.

### Lymphoblast cell culture

Patient lymphoblast cell lines were previously collected [28] and were grown as previously described [54]. The CLN6 patient lymphoblast line was from a male with Costa Rican ancestry harboring a homozygous mutation in exon 3 (c.214G>T, p.Glu72X) that predicts a prematurely truncated protein product [28].

### Immunostaining

For immunostaining of cultured cells and subsequent confocal microscopy, cells were seeded onto 18 mm diameter glass No. 1 coverslips (Fisher Scientific), inside a 12-well tissue culture petri dish, at a density of ∼4×10^4^ cells/well, and cells were grown overnight in Cbc media, at 33°C, 5% CO_2_. The following day, coverslips were fixed inside the well with either ice-cold 4% formaldehyde/PBS, pH 7.4, incubated for 20′ at room temperature, or with ice-cold methanol∶acetone (1∶1), for 10′ at −20°C, following by air drying, depending on the antibody. Following fixation, coverslips were rinsed with PBS, removed from the tissue culture dish, and processed for immunostaining, as previously described [20]. For buffer incubations, coverslips, set atop parafilm inside a large Petri dish, were overlaid with 100–300 µl of the appropriate solution, and aspiration from the coverslip edge was used to remove previous buffers. Following immunostaining, coverslips were mounted onto slides with ProLong® Gold antifade reagent with or without DAPI (Invitrogen), according to the manufacturer's recommendations. Nail polish-sealed coverslips were imaged on a Leica SP5 AOBS scanning laser confocal microscope (Leica Microsystems). Like-stained wild-type and homozygous mutant samples were mounted on the same microscope slide and were imaged in the same session, with identical settings.

### LysoTracker® and Endocytosis Assay

LysoTracker® staining and 10,000 molecular weight dextran-Alexa Fluor® 488 endocytic uptake was as previously described [32], but was adapted to a 96-well, high-content imaging format. Cerebellar cells were seeded into clear-bottomed, 96-well Costar® tissue culture plates (Corning Inc.) at a density of 5000 cells/well (100 µl volume). Following overnight incubation at 33°C, in a 5% CO_2_ humidified tissue culture incubator, the media was aspirated and exchanged for pre-warmed, fresh media containing 500 nM LysoTracker® DND-99 and 1 mg/ml dextran-Alexa Fluor® 488, using a multipipettor. Plates were immediately placed back in the tissue culture incubator. Following 30′ incubation, plates were fixed with ice-cold 4% formaldehyde in PBS, pH 7.4 on ice, for 20′. Wells were then rinsed with PBS, pH 7.4 five times, 10′ each. Following aspiration of the final PBS rinse, nuclei were counterstained with Hoechst dye for 5 minutes and rinsed twice in PBS, pH 7.4. Finally, 100 µl of PBS, pH 7.4 plus 0.5% sodium azide was added and plates were sealed and stored at 4°C. Plates were imaged on an ImagXpress Micro automated high-content imaging system (Molecular Devices), set to capture 3 sites per well, at 10× magnification. The Transfluor module of MetaXpress software (Molecular Devices) was used to determine the ‘mean vesicle count per cell’ from captured images. Images were segmented into nuclei and stained vesicle compartments using the ‘vesicles’ parameter in the Transfluor module. The ‘mean vesicle count per cell’ was determined from all nuclei captured in 3 images/well, and from 8 or more replicate wells per cell line. Poorly focused images were excluded from analysis. Red (LysoTracker®) and green (dextran-Alexa Fluor® 488) channels were analyzed separately. Significance was determined using a Student's T-test.

### Quantification of mitochondrial shape

Images of grp75 immunostained cells were collected on the same day with the same settings, and were then analyzed to quantify mitochondrial shape using ImageJ software (v1.44k for Macintosh; http://rsbweb.nih.gov/ij/). Images were first thresholded in ImageJ, then the ‘analyze particles’ function was used to segment and measure the mitochondrial circularity. A circularity index of ‘1’ indicates a perfect circle. At least 3 independent, random fields per coverslip, representing a total of ∼100–150 cells per line, were analyzed.

### Gene expression analysis

Gene expression experiments were carried out through the NIH Neuroscience Microarray Consortium, within the UCLA Center. Total RNA from three different cell lines per genotype was isolated at MGH using TRIzol reagent (Invitrogen), according to the manufacturer's recommendations. Prepared RNA samples were submitted to the UCLA DNA Microarray Facility (microarray.genetics.ucla.edu) for cDNA preparation and hybridization. All RNA samples passed quality checks on the Nanodrop (Thermo Scientific) and Agilent Bioanalyzer (Agilent Technologies). cDNA was prepared and hybridized to MOE 430 2.0 Affymetrix GeneChip® oligonucleotide microarrays, according to standard protocols at the UCLA Facility. Microarray data were corrected for backgrounds and normalized using gcRMA (R, 2.6.2; Biobase, 1.16.3; gcrma, 2.10.0).

The microarray data are MIAME compliant, and the raw data have been deposited in NCBI's Gene Expression Omnibus [55] and are accessible through GEO Series accession number GSE24368 (http://www.ncbi.nlm.nih.gov/geo/query/acc.cgi?acc=GSE24368).

Real-time qRT-PCR was performed to validate selected genes in the homozygous Cb*Cln3* ^Δex7/8^ and Cb*Cln6* ^nclf^ cell lines, according to standard procedures, using *Gapdh* and/or β-actin (*Actb*) as unchanged housekeeping gene controls (see Table S8 for specific primer information). From the same original total RNAs used for the array experiments, cDNA was generated from 1 µg RNA using SuperScript II reverse transcriptase (Invitrogen). Real-time PCR was performed using SYBR Green (Roche) according to the manufacturer's instructions and analyzed on a LightCycler 480 instrument (Roche) using the following thermocycling conditions: an initial denaturation step of 95°C for 5 minutes followed by 45 cycles of 95°C for 10 seconds, 56°C for 10 seconds, and 72°C for 10 seconds.

The DAVID Bioinformatics Resource 6.7 [56], [57] was used for further gene ontology analysis of significant probes.

For identification of significantly enriched pathways, we used sigPathway (1.6.0) (http://bioconductor.org/packages/release/bioc/html/sigPathway.html) [41]. Following analysis using sigPathway software, ‘NEk statistics’ and ‘NTk statistics’ were output as measures of how significant changes were for a given gene set (the higher the absolute value of the statistic, the greater its significance), and the direction of change (indicated by positive or negative statistics). ‘NEk statistics’ represented enrichment statistics based on phenotype permutations, and ‘NTk statistics’ represented gene set permutation-based test results (60,000 permutations). A false discovery rate cut-off of less than 0.01 was applied for both NTk and NEk statistics for each gene set, within a given genotype comparison (e.g. Cb*Cln6* ^nclf/nclf^ versus Cb*Cln6* ^+/+^ and Cb*Cln3* ^Δex7/8/Δex7/8^ versus Cb*Cln3* ^+/+^), and the total list of significantly changed gene sets (FDR<.01) are supplied in Tables S1 and S2. We considered the NTk statistics to have a greater reliability than the NEk statistics, given a limited number of unique permutations to construct a null distribution of test statistics. Therefore, in the summary tables presented in Tables 2 and 3, only the NTk values are shown. Both NTk and NEk values are shown in the supporting information tables (Tables S6 and S7).

### Subunit c accumulation assay

The subunit c accumulation assay was modified from that which has been previously described [20]. Cells were seeded onto 100 mm petri dishes at a density of 2×10^5^ cells/plate and subsequently incubated at 33°C, 5% CO_2_ for between seven and ten days. Separate plates for each cell line were maintained at sub-confluent density, as described above, for use as un-aged controls, which do not show significant subunit c accumulations in this assay.

For immunostaining, confluency aged or un-aged control cells were trypsinized and replated onto 18 mm No. 1 glass coverslips inside a 12-well tissue culture plate at a density of 8×10^4^ cells/well. Replated cells were then incubated overnight at 33°C, 5% CO_2_, prior to fixation (methanol∶acetone, 1∶1) and immunostaining, which were carried out as described above.

### ATP assay

Un-aged control and mutant cells were trypsinized and replated into clear-bottomed, 96-well Costar® tissue culture plates (Corning Inc.) at a density of 10, 000 cells/well (100 µl volume). Total cellular ATP levels were assayed using the CellTiter-GLO® Luminescent Cell Viability kit (Promega), according to the manufacturer's recommendations and as previously described [20]. Experiments conducted on the set of Cb*Cln3* cell lines (i.e. wild-type, heterozygous, and homozygous lines) were performed at the same time, and those on Cb*Cln6* cell lines were performed at the same time, but independently from the Cb*Cln3* cell lines. To enable comparison across experiments, absolute RLUs were normalized to the respective wild-type numbers.

## Supporting Information

Figure S1 **Marker immunostaining of Cb** ***Cln6*** **^nclf^ cerebellar neuronal precursor cells.** Representative micrographs of nestin- (green) and GFAP-(red) immunostained wild-type (Cb*Cln6* ^+/+^), heterozygous (Cb*Cln6* ^+/nclf^), and homozygous (Cb*Cln6* ^nclf/nclf^) neuronal precursor cell lines are shown. Selected clones were further confirmed as positive or negative for the markers by immunoblot analysis (not shown). 20× magnification.
(TIF)

Figure S2 **Quality control of the Cb** ***Cln3*** **^Δex7/8^ and Cb** ***Cln6*** **^nclf^ cell gene expression datasets using principal components analysis (PCA).** PCA plots for Cb*Cln6* ^nclf^ cells (top row, red circles) and Cb*Cln3* ^Δex7/8^ cells (bottom row, blue circles) are shown. In all plots, closed circles represent data from mutant cells and open circles represent data from wild-type cells. As expected, PCA plots for the entire gcrma-normalized datasets (‘Total Probes’) show good separation by genotype, but also some variation among biological replicates, which most likely arose from the original derivation of the cell lines, which were from different mouse pups of the same genotype. The use of biological replicates from independent animals for our gene expression study was desirable in order to achieve our goal of capturing the gene expression variation that was a consequence of the genetic mutation. To further explore the variation in our datasets, we performed additional PCA analyses on the most variable probes (‘Top 1000 Variable Probes’), the most variable significant probes (‘Significant Probes’), and the most variable non-significant probes (‘Non-significant Probes’). The PCA plots for the ‘Top 1000 Variable Probes’ were highly similar to the ‘Total Probes’ PCA plots, demonstrating that restricting our analysis to a smaller set of probes did not dramatically alter the variation structure among the samples. However, PCA analysis of the top significant probes for each comparison (106 probes in Cb*Cln6* ^+/+^ versus Cb*Cln6* ^nclf/nclf^ cells, and 110 probes in Cb*Cln3* ^+/+^ versus Cb*Cln3* ^Δex7/8/Δex7/8^ cells) showed good separation by genotype, and the biological replicates were tightly overlapping on PC1. Conversely, the top non-significant probes did not produce strong separation by genotype or biological replicate in the PCA plots (for consistency, the quantity of probes used was kept the same as for the signficant probes analysis). Therefore, these data suggested that PC1 in the PCA using all probes (‘Total Probes’) captured genotype-correlated variance, and that non-genotype-correlated variance (i.e. PC2) was effectively removed in our significantly altered probes.
(TIF)

Figure S3 **PCA analysis of significant probes across the Cb** ***Cln3*** **^Δex7/8^ and Cb** ***Cln6*** **^nclf^ cell datasets.** PCA plots for Cb*Cln6* ^nclf^ cells (top row) and Cb*Cln3* ^Δex7/8^ cells (bottom row) are shown, representing significant probes (left graphs), and significant probes from the opposing NCL genotype datasets (right graphs). For relevant comparisons of plots, we used the same scale for each genotype. Note that the significant probes (p<.01, fold-change>1.5) from the Cb*Cln6* ^nclf^ cells dataset, plotted for wild-type Cb*Cln6* ^+/+^ (open red circles) and homozygous mutant Cb*Cln6* ^nclf^ cells (closed red circles), clearly separated the wild-type and mutant genotypes, and that the biological replicates were strongly overlapping (top left panel). The same was true of the significant probes (p<.01, fold-change>1.5) from the Cb*Cln3* ^Δex7/8^ cells dataset, plotted for wild-type (open blue circles) and homozygous mutant (closed blue circles) Cb*Cln3* ^Δex7/8^ cells (bottom left panel). To the contrary, the significant probes from the unrelated genotype dataset, plotted for wild-type (open circles) and homozygous mutant (closed circles) cells, did not strongly distinguish the wild-type from mutant for either the Cb*Cln6* ^nclf^ cells (top right) or the Cb*Cln3* ^Δex7/8^ cells (bottom right).
(TIF)

Figure S4 **Scatterplot analysis of NTk Statistics for Cb** ***Cln3*** **^Δex7/8^ and Cb** ***Cln6*** **^nclf^ significant pathways.** Scatterplots of Cb*Cln3* ^Δex7/8^ versus Cb*Cln6* ^nclf^ NTk statistics (NTk Stat) are shown for the significant pathways from the Cb*Cln3* ^Δex7/8^ dataset (left), and for the significant pathways from the Cb*Cln6* ^nclf^ dataset, identified by the sigPathway program. There was a negative correlation between the significant pathways identified in the homozygous Cb*Cln3* ^Δex7/8^ and Cb*Cln6* ^nclf^ cells (Pearson correlation coefficients and significance values are shown), suggesting some overlap in the pathways affected by the *Cln3* ^Δex7/8^ and *Cln6* ^nclf^ mutations, but that the direction of change in the pathways was typically different.
(TIF)

Table S1 **Significant probes in the Cb** ***Cln3*** **^Δex7/8^ dataset.** The Affymetrix probes (Probe ID), with significant (p<0.01, Student's t-test) expression level differences in the Cb*Cln3* ^Δex7/8/Δex7/8^ cells, versus the Cb*Cln3* ^+/+^ cells are shown, along with the corresponding gene symbols and titles. Mean expression values were determined across three biological replicates per genotype, and the standard deviation (SD) for the replicates is shown. Log2 fold-change (‘Fold Change’) is also shown. A cut-off was applied of greater than 1.5-fold change, to arrive at the list of significant probes.
(XLS)

Table S2 **Significant probes in the Cb** ***Cln6*** **^nclf^ dataset.** The Affymetrix probes (Probe ID), with significant (p<0.01, Student's t-test) expression level differences in the Cb*Cln6* ^nclf/nclf^ cells, versus the Cb*Cln6* ^+/+^ cells are shown, along with the corresponding gene symbols and titles. Mean expression values were determined across three biological replicates per genotype, and the standard deviation (SD) for the replicates is shown. Log2 fold-change (‘Fold Change’) is also shown. A cut-off was applied of greater than 1.5-fold change, to arrive at the list of significant probes.
(XLS)

Table S3 **qRT-PCR to validate the relative expression of selected genes in Cb** ***Cln6*** **^nclf^ and Cb** ***Cln3*** **^Δex7/8^ cells.** Gene expression levels were normalized to *Gapdh* or *Actb* housekeeping gene expression levels. Representative means measured across at least triplicate qRT-PCR reactions are shown. P-values to determine the significance of the expression level differences in wild-type versus mutant cells were calculated using a student's t-test. Primers used are shown in Table S8. n.d. = not determined. *Tagln2* gene expression was not determined for the Cb*Cln3* set of cells because this gene change was only identified in the Cb*Cln6* dataset.
(XLS)

Table S4 **DAVID gene ontology analysis of significant Cb** ***Cln3*** **^Δex7/8^ probes.** The significantly enriched (p<0.01) gene ontology terms (GOTERM/Term) from the Biological Process and Cellular Component categories are shown, determined through analysis of the significant Cb*Cln3* ^Δex7/8/Δex7/8^ probes in DAVID Bioinformatics Resources 6.7. The number of probes (and the % of the total) that were represented by the GO term (Probe Count) is indicated, and the Probe-IDs are listed (Probes). The relative enrichment value (Fold Enrichment) is also shown.
(XLS)

Table S5 **DAVID gene ontology analysis of significant Cb** ***Cln6*** **^nclf^ probes.** The significantly enriched (p<0.01) gene ontology terms (GOTERM/Term) from the Biological Process and Cellular Component categories are shown, determined through analysis of the significant Cb*Cln6* ^nclf/nclf^ probes in DAVID Bioinformatics Resources 6.7. The number of probes (and the % of the total) that were represented by the GO term (Probe Count) is indicated, and the Probe-IDs are listed (Probes). The relative enrichment value (Fold Enrichment) is also shown.
(XLS)

Table S6 **Significantly altered gene sets identified by sigPathway analysis of the Cb** ***Cln3*** **^Δex7/8^ dataset.** The significantly altered (q-value<0.01) gene sets, and associated statistics details, are shown for the Cb*Cln3* ^Δex7/8^ cells. For comparison, the statistics for these same gene sets are shown for the Cb*Cln6* ^nclf^ cells. For a complete description of the NTk and NEk value determinations, see Materials and Methods.
(XLS)

Table S7 **Significantly altered gene sets identified by sigPathway analysis of the Cb** ***Cln6*** **^nclf^ dataset.** The significantly altered (q-value<0.01) gene sets, and associated statistics details, are shown for the Cb*Cln6* ^nclf^ cells. For comparison, the statistics for these same gene sets are shown for the Cb*Cln3* ^Δex7/8^ cells. For a complete description of the NTk and NEk value determinations, see Materials and Methods.
(XLS)

Table S8 **Primers used for qRT-PCR validation of expression array hits.** For each selected gene target, primers used for qRT-PCR experiments are shown, with the sequence in the 5′ to 3′ orientation.
(DOC)
