# Supplementary figures and images for: Distinct Early Molecular Responses to Mutations Causing vLINCL and JNCL Presage ATP Synthase Subunit C Accumulation in Cerebellar Cells
Source: PLoS One. 2011 Feb 17;6(2):e17118. doi: 10.1371/journal.pone.0017118 (PMC3040763; doi:10.1371/journal.pone.0017118)

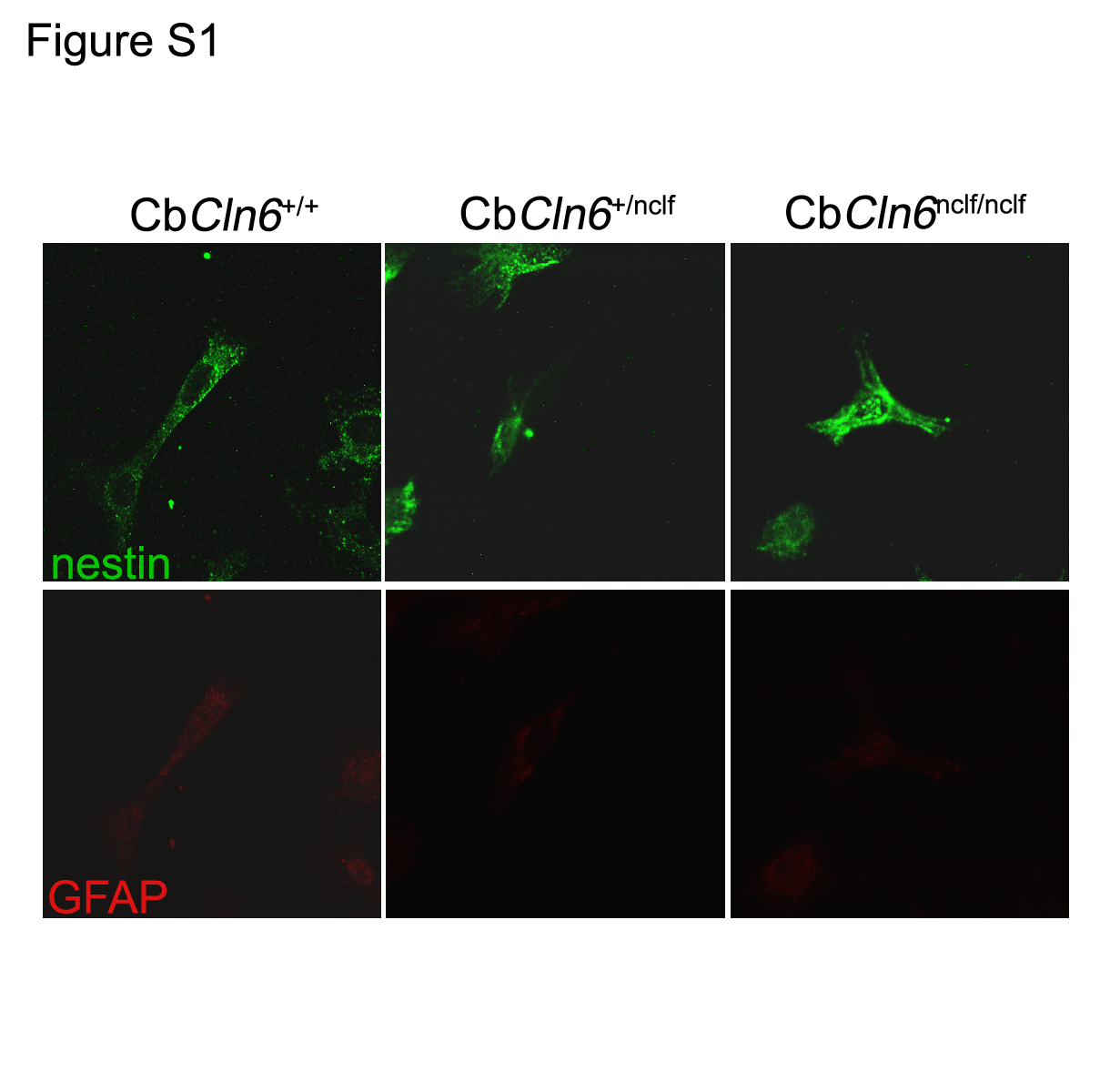

Supplement: Figure S1 — Marker immunostaining of Cb Cln6 nclf cerebellar neuronal precursor cells. Representative micrographs of nestin- (green) and GFAP-(red) immunostained wild-type (CbCln6 +/+), heterozygous (CbCln6 +/nclf), and homozygous (CbCln6 nclf/nclf) neuronal precursor cell lines are shown. Selected clones were further confirmed as positive or negative for the markers by immunoblot analysis (not shown). 20× magnification. (TIF) [file pone.0017118.s001.tif]

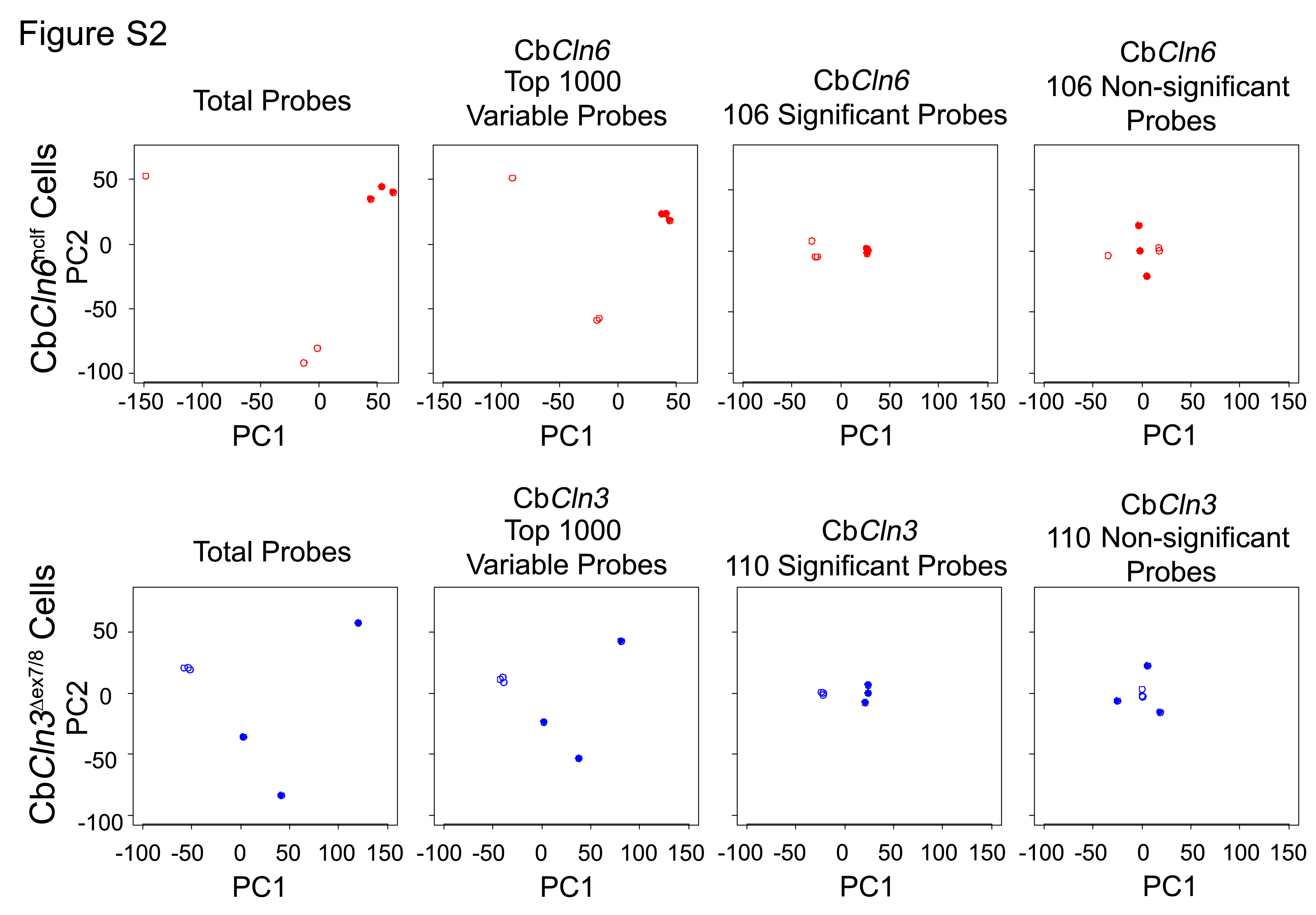

Supplement: Figure S2 — Quality control of the Cb Cln3 Δex7/8 and Cb Cln6 nclf cell gene expression datasets using principal components analysis (PCA). PCA plots for CbCln6 nclf cells (top row, red circles) and CbCln3 Δex7/8 cells (bottom row, blue circles) are shown. In all plots, closed circles represent data from mutant cells and open circles represent data from wild-type cells. As expected, PCA plots for the entire gcrma-normalized datasets (‘Total Probes’) show good separation by genotype, but also some variation among biological replicates, which most likely arose from the original derivation of the cell lines, which were from different mouse pups of the same genotype. The use of biological replicates from independent animals for our gene expression study was desirable in order to achieve our goal of capturing the gene expression variation that was a consequence of the genetic mutation. To further explore the variation in our datasets, we performed additional PCA analyses on the most variable probes (‘Top 1000 Variable Probes’), the most variable significant probes (‘Significant Probes’), and the most variable non-significant probes (‘Non-significant Probes’). The PCA plots for the ‘Top 1000 Variable Probes’ were highly similar to the ‘Total Probes’ PCA plots, demonstrating that restricting our analysis to a smaller set of probes did not dramatically alter the variation structure among the samples. However, PCA analysis of the top significant probes for each comparison (106 probes in CbCln6 +/+ versus CbCln6 nclf/nclf cells, and 110 probes in CbCln3 +/+ versus CbCln3 Δex7/8/Δex7/8 cells) showed good separation by genotype, and the biological replicates were tightly overlapping on PC1. Conversely, the top non-significant probes did not produce strong separation by genotype or biological replicate in the PCA plots (for consistency, the quantity of probes used was kept the same as for the signficant probes analysis). Therefore, these data suggested that PC1 in the PCA using all probes ( [file pone.0017118.s002.tif]

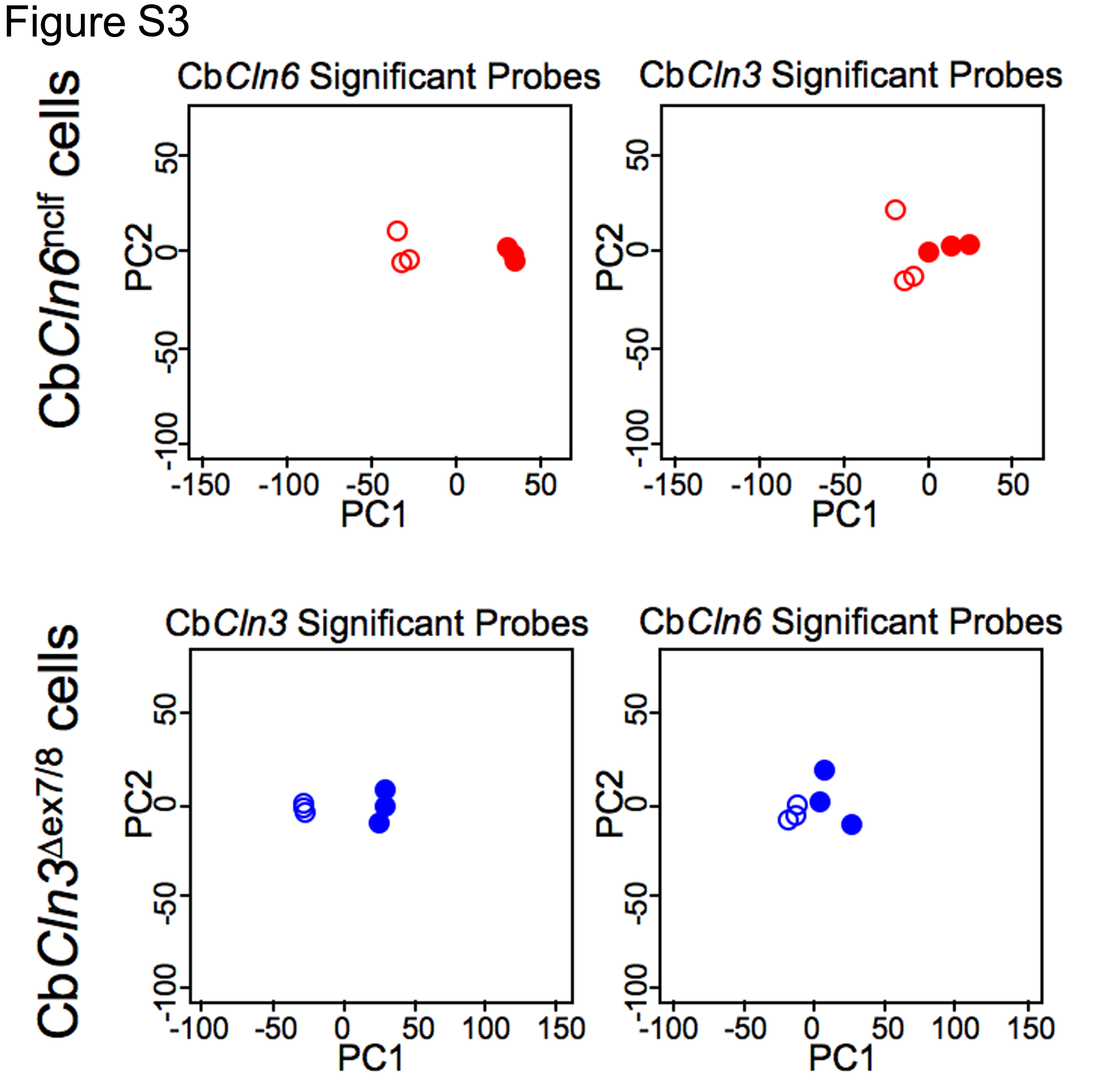

Supplement: Figure S3 — PCA analysis of significant probes across the Cb Cln3 Δex7/8 and Cb Cln6 nclf cell datasets. PCA plots for CbCln6 nclf cells (top row) and CbCln3 Δex7/8 cells (bottom row) are shown, representing significant probes (left graphs), and significant probes from the opposing NCL genotype datasets (right graphs). For relevant comparisons of plots, we used the same scale for each genotype. Note that the significant probes (p<.01, fold-change>1.5) from the CbCln6 nclf cells dataset, plotted for wild-type CbCln6 +/+ (open red circles) and homozygous mutant CbCln6 nclf cells (closed red circles), clearly separated the wild-type and mutant genotypes, and that the biological replicates were strongly overlapping (top left panel). The same was true of the significant probes (p<.01, fold-change>1.5) from the CbCln3 Δex7/8 cells dataset, plotted for wild-type (open blue circles) and homozygous mutant (closed blue circles) CbCln3 Δex7/8 cells (bottom left panel). To the contrary, the significant probes from the unrelated genotype dataset, plotted for wild-type (open circles) and homozygous mutant (closed circles) cells, did not strongly distinguish the wild-type from mutant for either the CbCln6 nclf cells (top right) or the CbCln3 Δex7/8 cells (bottom right). (TIF) [file pone.0017118.s003.tif]

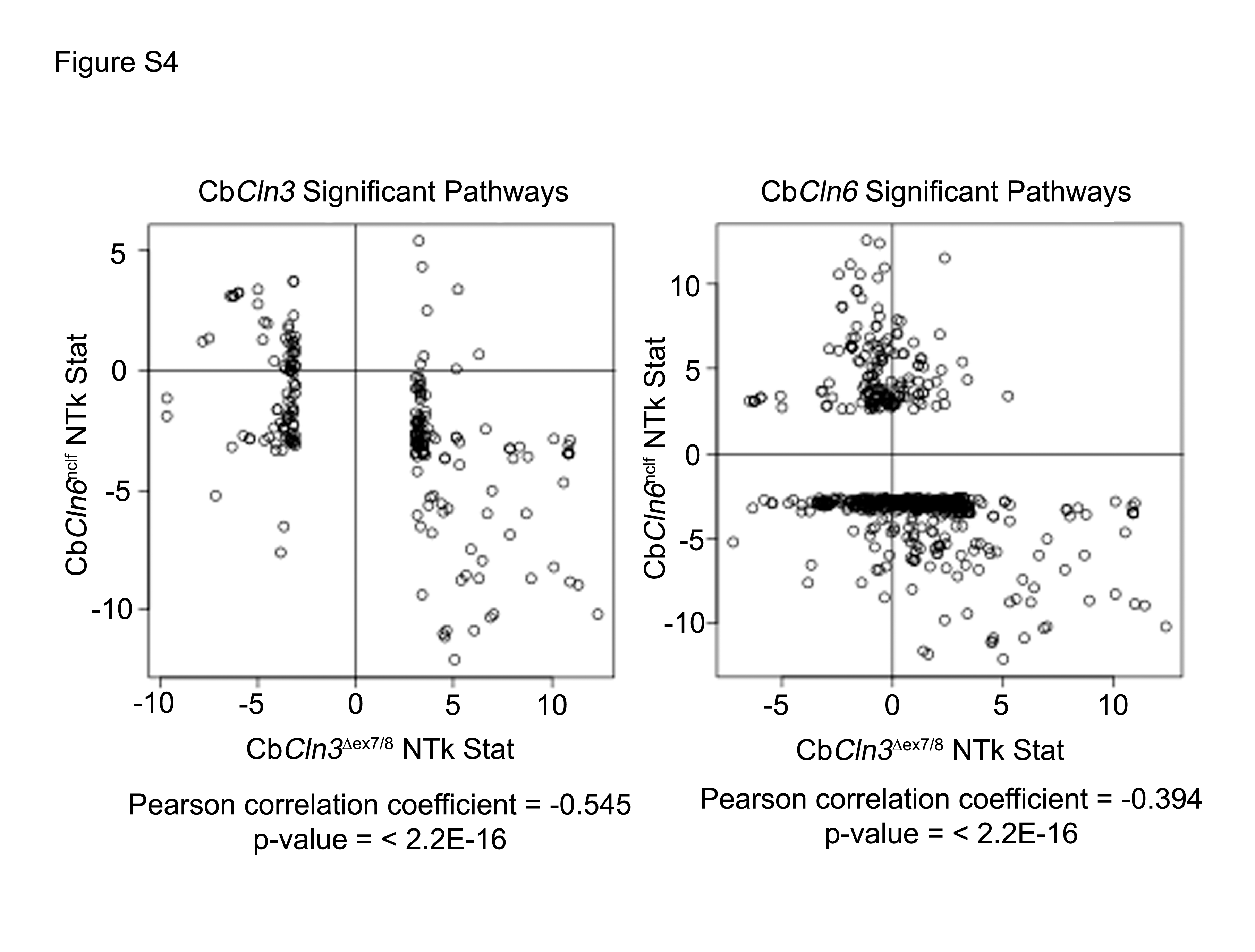

Supplement: Figure S4 — Scatterplot analysis of NTk Statistics for Cb Cln3 Δex7/8 and Cb Cln6 nclf significant pathways. Scatterplots of CbCln3 Δex7/8 versus CbCln6 nclf NTk statistics (NTk Stat) are shown for the significant pathways from the CbCln3 Δex7/8 dataset (left), and for the significant pathways from the CbCln6 nclf dataset, identified by the sigPathway program. There was a negative correlation between the significant pathways identified in the homozygous CbCln3 Δex7/8 and CbCln6 nclf cells (Pearson correlation coefficients and significance values are shown), suggesting some overlap in the pathways affected by the Cln3 Δex7/8 and Cln6 nclf mutations, but that the direction of change in the pathways was typically different. (TIF) [file pone.0017118.s004.tif]
