# Supplementary material for: Distinct Early Molecular Responses to Mutations Causing vLINCL and JNCL Presage ATP Synthase Subunit C Accumulation in Cerebellar Cells
Source: PLoS One. 2011 Feb 17;6(2):e17118. doi: 10.1371/journal.pone.0017118 (PMC3040763; doi:10.1371/journal.pone.0017118)
Supplement: Table S8 — Primers used for qRT-PCR validation of expression array hits. For each selected gene target, primers used for qRT-PCR experiments are shown, with the sequence in the 5′ to 3′ orientation. (DOC) [file pone.0017118.s012.doc]

**Table S8:** **Primers used for qRT-PCR validation of expression array hits**

| **Target** | **Forward Primer*** | **Reverse Primer*** |
| --- | --- | --- |
| mCln3 | GGTTTCTGGATCTTGGGTCT | GTGGAGATGGAGTTGCAGTC |
| mCln6 | CCTTCCATCTTGACCTCTGG | GAAGGGCGTGATGATGTTGT |
| mSnf8 | AGCAAGCACAAGCAAGAGAT | CACCTGCTGATGTAGCTCCT |
| mFscn1 | TTGACATCGAGTGGTGTGAC | TTCCAACTGGAAGACATCGT |
| mTagln2 | CCTTGGCTAGGAGTCAGAGA | CCCAGACGTGTGGTAAGTACA |
| mGapdh | TAACATCAAATGGGGTGAGG | GTTCACACCCATCACAAACA |
| mActb | GACGGCCAGGTCATCACTAT | ATGCCACAGGATTCCATACC |

*Primer sequences are shown 5' to 3'.
